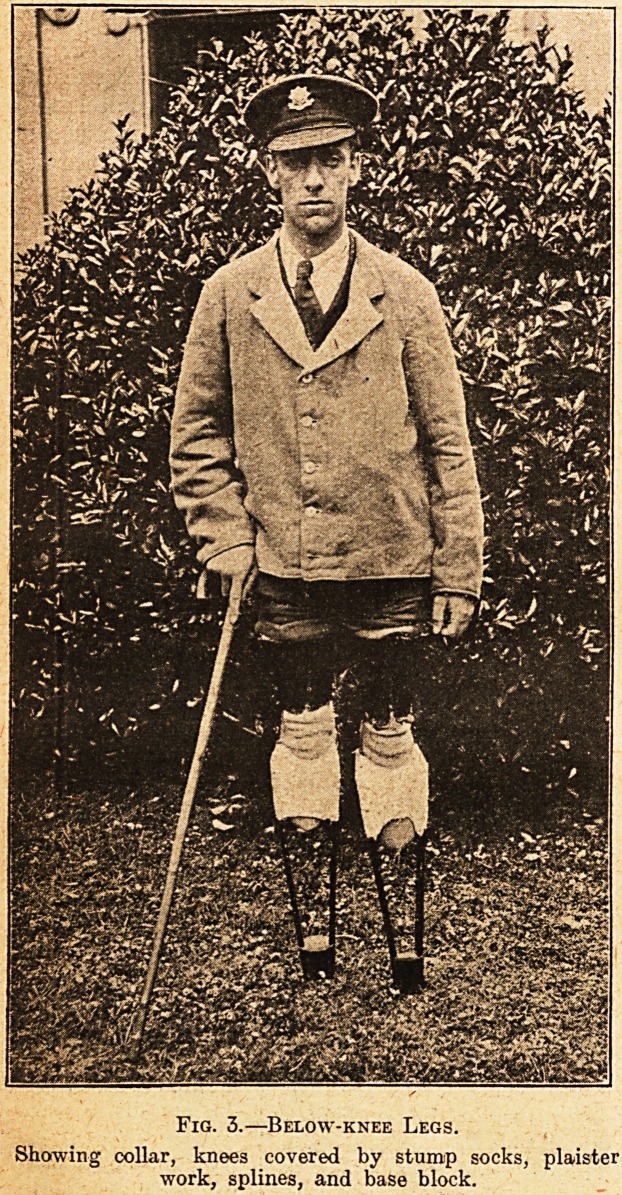# As Made at the Pavilion General Hospital, Brighton

**Published:** 1918-07-20

**Authors:** 


### TEMPORARY ARTIFICIAL LEGS

### As Made at the Pavilion General Hospital, Brighton.

### VIII.—Amputation Below the Knee—Syme's Excluded.

A "BELOW-THE-KNEE AMPUTATION" is fitted with a temporary leg of a pattern that allows movement of the knee joint. This leg suits all stumps except cases of Syme's amputation and the amputations similar to Syme's, for which a special leg is fashioned in papier-mâché.

The base block, the indiarubber pad, and the

FIG. 1.—BELOW-THE-KNEE STUMP LEG.

A. The ironwork, hinged, drilled for screws, still straight. B. Leg ready for collar. C. Collar. D. Leg completed. E. The tool devised to wrench and twist ironwork.

splines are made as in the above-knee cases, except that the splines are shorter. The splines, as first cut, vary between 18 and 22 inches. The measurement is taken from the condyles of the femur of the sound leg to the ground whilst the man is seated and is wearing his boot. The splines on this measurement are two or three inches longer than they will be in the end.

Four pieces of iron are cut from $\frac{3}{4}$ in. by $\frac{3}{32}$ in. bars. Each is straight, $8\frac{1}{2}$ in. long. Two are drilled with three holes for screws. One hole has its centre $\frac{1}{2}$ in. from one end, the other two have their centres 2 in. and 4 in. from the centre of the first hole. A fourth hole, for a rivet, is drilled at the other end of the piece. The other two pieces are drilled each close to an end. Each of the pieces with four holes is riveted by a loose rivet to one of the pieces with only one hole. Thus a hinge which is to be at the knee joint is formed.

This ironwork is fitted to the inner sides of the splines, so that the hinge is just above the top of the spline and the three screw holes over the woodwork, and fastened to the woodwork by screws. Then about 2 in. of each spline are cut off, the new end tapered off neatly, and the outer side of the spline slightly grooved with a file to enable the plaister to get a firm grip.

#### MAKING THE "COLLAR."

Meanwhile the "collar" is being fashioned. It is made to fit the thigh above the knee. It consists of a piece of fibre about 7 in. by 14 in. The length and breadth vary with the thigh. This piece of fibre is rounded off at the corners and scooped out in the central part of the lower edge till the centre part is about 5 in. wide. Thus it is kidney-shaped. Along the two ends five or six eyelet holes are made and eyeleted as a boot. A felt lining is stitched on

FIG. 2.—TWO LEGS COMPLETE.
One for thigh stump, one for below-knee stump.

so that it projects slightly round the edge of the fibre. Laces are provided. The collar laces at the front.

The man, sitting on a chair, has a collar fastened on to his thigh. The splines are then put in the position relative to the stump in which they will

Previous articles appeared on July 6 and 13, p. 373.

**Temporary Artificial Legs**—(*continued*).

be when the leg is completed and the necessary adjustment of the ironwork is noted. The ironwork requires to be thrown outwards. This is done with a "twisting wrench," a special tool devised here, but a screw wrench does nearly as well. By wrenching outwards the right amount of space is given between the knee and the ironwork, and by wrenching usually inwards the upper iron is made to rest flatly on the collar. These bends will vary

FIG. 3.—BELOW-KNEE LEGS.
Showing collar, knees covered by stump socks, plaister work, splines, and base block.

with every case, and are a part of the work that experience and practice greatly improve.

FIXING THE PLAISTER.

The framework is now ready for the plaister. The man sits on a chair; the collar is on his thigh; the upper irons are fixed to the collar by a strap; the hinge is bent to about a right angle; the man's knee is straightened so that the stump projects straight out. The operator, kneeling in front of him, greases the stump, covers any soft scars or unhealed areas or excessively hairy parts with oil silk, fills any hollows with cotton-wool, and places a layer of cotton-wool over the shin-bone and over the head of the fibula. The plaister bandages are smoothly applied till five or six layers are in place.

The lower end of the frame is now raised till the splines are parallel to the long axis of the stump, not of the thigh, and figure-of-eight strips are passed round one after the other, beginning with the inside spline, till the mould is firmly fixed to the splines. The hollows at each side of the splines are now filled up with cotton-wool and plaister till smoothness of curve is acquired, and then a few more layers of bandage are applied to give finish and neatness. A final smoothness is given by smearing on plaister with the hands.

FITTING THE MOULD.

The mould is now slipped off the stump and left to dry till next day, when the man comes to be fitted. The fitting consists in putting on the leg and marking with pencil and cutting away bit by bit till the upper edge fits neatly and comfortably. The lower edge is trimmed in the same manner and both edges bound off neatly. The mould is put on again, and the man stands up with it on and the collar on. The ironwork is applied to the collar, and, by eye, places for rivet holes are marked on each piece of iron. These holes are then drilled, the iron is applied to the collar again, and corresponding marks to the holes made on the collar. The collar is taken off, the holes punched, rivets are passed from within outwards, and clinched. The leg is complete. A sock is put on the stump, the leg put on, and the man walks off with more or less steadiness according to his nerve.

G. T. K. MAURICE, Colonel A. M. S.,
O.C., Pavilion General Hospital.

*(To be continued.)*

## Figures and Tables

**Fig. 1. f1:**
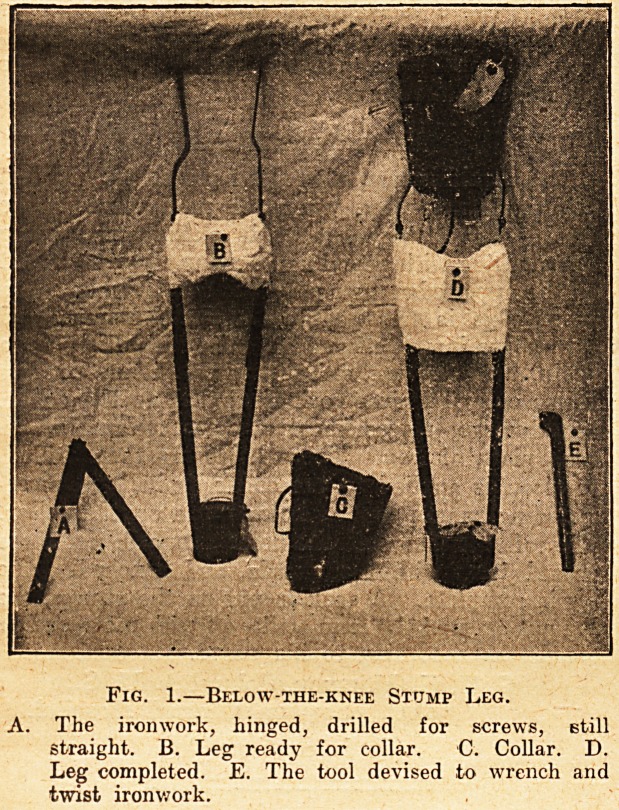


**Fig. 2. f2:**
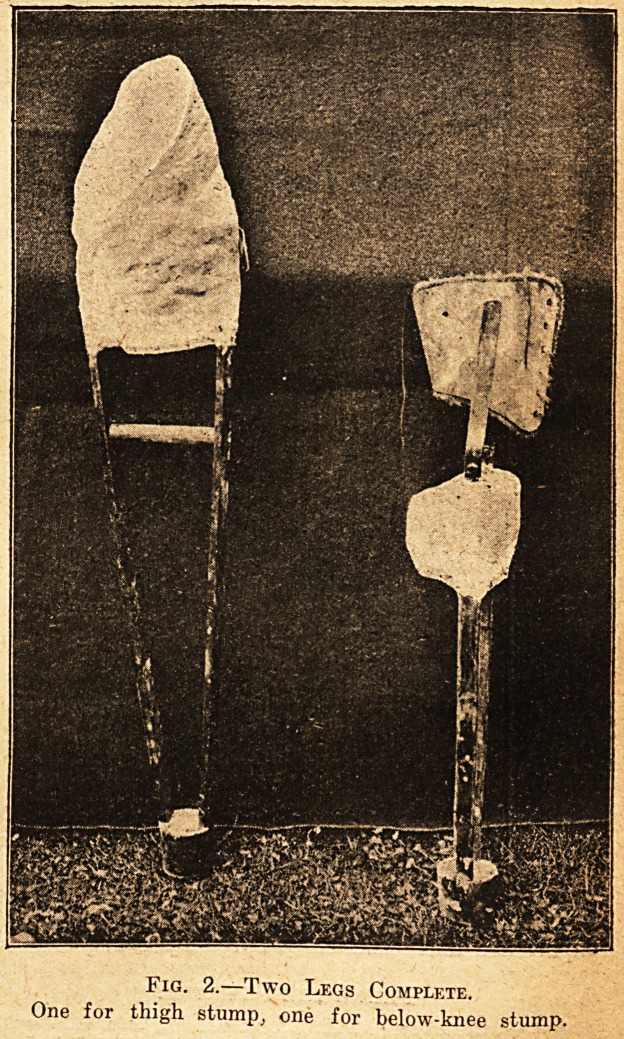


**Fig. 3. f3:**